# Waist-hip ratio is an independent predictor of moderate-to-severe OSA in nonobese males: a cross-sectional study

**DOI:** 10.1186/s12890-022-01886-3

**Published:** 2022-04-22

**Authors:** Yan Wang, Lusi Mao, Xiaolei Zhang

**Affiliations:** 1 Department of Pulmonary and Critical Care Medicine, Center of Respiratory Medicine, China–Japan Friendship Hospital, 2 East Yinghua (Cherry Blossom) Road, Hepingli, Chaoyang District, Beijing, 100029 China; 2 Graduate School of Peking Union Medical College, Chinese Academy of Medical Science and Peking Union Medical College, Beijing, China; 3 Capital Medical University, Beijing, China; 4 Peking University Health Science Center, Beijing, China

**Keywords:** Waist-hip ratio, Moderate-to-severe OSA, Nonobese patients

## Abstract

**Background:**

Adiposity is a well-established risk factor for obstructive sleep apnea (OSA), but whether a combination of preferable anthropometric measurements may improve the accuracy of detecting OSA is unknown. This study aimed to explore the accuracies of the waist-hip ratio (WHR) in conjunction with the body mass index (BMI) when identifying the severity of OSA.

**Design:**

A total of 2012 participants in the China-Japan Friendship Hospital from January 2018 to December 2019 underwent anthropometric measurements and an overnight home sleep test (HST). The 244 subjects who met the criteria for obstructive sleep apnea (apnea–hypopnea index (AHI) ≥ 5 events/hour) were divided into four groups: Group A (55 patients with WHR ≥ 0.9 and BMI ≥ 28 kg/m^2^); Group B (12 patients with WHR < 0.9 and BMI ≥ 28 kg/m^2^); Group C (69 patients with WHR ≥ 0.9 and BMI < 28 kg/m^2^); and group D (108 patients with WHR < 0.9 and BMI < 28 kg/m^2^).

**Results:**

The AHI, apnea index (AI), hypopnea index (HI), and oxygen desaturation index (ODI) were significantly different among the 4 groups (*p* < 0.05). The WHR was positively correlated with AHI (r = 0.22, *p* < 0.001), AI (r = 0.270, *p* = 0.004), and ODI (r = 0.286, *p* = 0.0022) and negatively correlated with lowest oxygen pulse saturation (LSpO_2_) (r = 0.246, *p* = 0.008) only in nonobese patients. Moreover, the WHR was found to be a screening marker for moderate-to-severe OSA in Group D (*p* < 0.05). When used to identify severe OSA in Group D, the WHR cut-off point of 0.873 yielded a sensitivity of 65% and specificity of 56% (*p* < 0.05).

**Conclusion:**

In nonobese male OSA patients, WHR is a moderate screening marker for moderate-to-severe OSA and an independent risk factor for OSA severity.

**Supplementary Information:**

The online version contains supplementary material available at 10.1186/s12890-022-01886-3.

## Introduction

Obstructive sleep apnea (OSA) is a respiratory disease characterized by repetitive airway collapse during sleep along with a cession (apnea)/reduction of airflow (hyponea) [1]. Given its high prevalence in obese individuals and the current global rise of obesity, OSA is estimated to be as prevalent as diabetes in developed countries [2]. Over 50% of obese people suffer from OSA, whether they are male or female [3, 4]. The prevalence of OSA is as high as 98% in morbidly obese patients [5]. Sleep disorder is attributable to excess weight (body mass index (BMI) ≥ 25 kg/m^2^) in 41% of mild patients and in 58% of moderate or severe patients [6]. A 10% increase in weight gain predicted a 32% increase in the apnea–hypopnea index (AHI), while a 10% weight loss predicated a 26% reduction in AHI. In addition, a 10% increase in weight predicted a sixfold increase in the risk of moderate-to-severe OSA [7].

With the improved quality of life, OSA is becoming increasingly common; however, since the diagnosis of OSA relies on laboratory polysomnography (PSG) or home sleep test (HST), the undiagnosed rate of OSA remains high. Effort is required to further shed light on the relationship between OSA and anthropometric measures. BMI has traditionally been the chosen surrogate method used to determine excessive body fat; however, as it is a weight-for-height measure, BMI is unable to distinguish fat deposits. As the amount of adipose tissue adjacent to the pharyngeal airway and in the intraperitoneal space is associated with AHI but not BMI, although higher BMI patients with OSA have more visceral fat, which is associated with severity, accumulative evidence suggests that visceral fat and central obesity are more sensitive predictive parameters for OSA and its severity [8, 9]. Therefore, BMI is not a good indicator for OSA.

Excessive central fats (abdominal and visceral fat) are associated with deleterious metabolism [10] and peripheral fats (hip and gluteal-femoral fat) contribute to metabolic protection [11]. An abnormal accumulation of adipose tissue is located in the tongue, soft palate, and uvula of OSA patients, and this increases the mechanical load to diminish the lumen of the airway and facilitate the collapse of the upper airway. Therefore, it is essential to determine the accumulation of central fats. Several radiologic methods have been used to measure central obesity, such as nuclear magnetism, ultrasonic examination, and positron emission tomography-computed tomography (PET-CT). Compared to the expensive and time-consuming methods listed above; the waist-to-hip ratio (WHR) might be the most pragmatic clinical measure of central obesity. The close correlation between the WHR and OSA severity has been extensively studied [12, 13]. However, inferences from these studies are limited, because many have been restricted to obese patients, or have focused on European and American people. It also not clear whether the relationships between the WHR and OSA severity observed in these studies could be simply explained by the BMI. To address these limitations, this study was conducted on admitted patients to determine the relationship between the WHR and OSA in Chinese people.

## Materials and methods

### Subjects

We enrolled 2012 patients with consecutive suspected OSA symptoms (excessive daytime sleepiness, loud snoring, or witnessed apnea) who were referred to a sleep laboratory in the Sleep Center of China-Japan Friendship Hospital from January 2018 to December 2019. This study was approved by the Institutional Ethics Committee of the China-Japan Friendship Hospital.

The exclusion criteria were as follows: (1) age less than 18 years; (2) history of OSA diagnosis or treatment; and (3) severe comorbid diseases, such as hypertension, diabetes or cardiovascular diseases. Patients were excluded due to hypertension if it was listed in their medical history, if their systolic blood pressure was 140 mmHg or higher or if the diastolic blood pressure was 90 mmHg or higher at the time of their visit. There was no ambulatory blood pressure monitoring (ABPM) performed in the study. Further exclusion criteria included the following: (4) recorded total sleep time (TST) < 4 h; (5) sleep disorders other than OSA, such as central sleep apnea or narcolepsy; (6) significant abnormal maxillofacial structures via upper airway CT; and (7) non-male patients. Finally, there were a total of 244 eligible subjects in our study (Additional file 1).

### Sleepiness evaluation and anthropometric measurements

Before the overnight HST, all participants were asked to complete the Epworth Sleepiness Scale (ESS) to assess daytime sleepiness. Briefly, the waist circumference (WC) was measured using an inelastic 150-cm tape at the midpoint between the inferior edge of the costal border and the iliac crest in the mid-axillary line, and the hip circumference (HC) was measured at the maximum posterior protrusion of the gluteus muscles. We measured the patient height to the nearest 0.1 cm. The body weight was measured with an electronic scale with a maximum capacity of 200 kg. BMI was calculated by dividing the participant's weight in kilograms by the square of their height in meter (kg/m^2^). The WHR was calculated by dividing WC (cm) by HC (cm).

### HST

The sleep studies were performed using a Nox T3 sleep monitor (Nox Medical, GA, USA), a standardized level-3 portable diagnostic device, as previously described [14]. All studies were manually scored by a sleep medicine expert according to the American Academy of Sleep Medicine 2012 [15]. Respiratory variables including chest and abdominal wall movements, nasal airflow and pressure, and oxygen saturation were recorded. Apnea was defined as a ≥ 90% decrease in airflow from the pre-event baseline level for over 10 s. Hypopnea was defined by a ≥ 30% drop in airflow lasting at least 10 s with a ≥ 3% SpO_2_ drop. The sum of apnea and hypopneas per hour determined the AHI. The number of apnea events per hour was defined as the apnea index (AI), while the hypopnea index (HI) was calculated as the total number of hypopnea events divided by the TST. The lowest oxygen pulse saturation (LSpO_2_) was defined as the lowest oxygen pulse saturation during sleep. The oxygen desaturation index (ODI) was considered the average number of 3% desaturation episodes from the baseline per hour of recording.

### Grouping

Since all the enrolled subjects were of Chinese ethnicity, the Chinese version of the BMI category for obesity was applied. Based on the HST, BMI and anthropometric measurement results, the subjects with OSA (AHI ≥ 5 events/hour) were further divided into four groups: individuals with central obesity (Group A; 55 patients with WHR ≥ 0.9 and BMI ≥ 28 kg/m^2^); individuals with noncentral obesity (Group B; 12 patients with WHR < 0.9 and BMI ≥ 28 kg/m^2^); nonobese individuals with central fat (Group C; 69 patients with WHR ≥ 0.9 and BMI < 28 kg/m^2^); and nonobese individuals (Group D; 108 patients with WHR < 0.9 and BMI < 28 kg/m^2^).

### Statistical analysis

The statistical analysis was carried out using SPSS 20.0 software. The continuous data were expressed as the mean ± standard deviation (SD). For categorical data, frequencies and percentages were reported. The mean differences among groups were tested using one-way ANOVA. Pearson's correlation coefficients were examined between sleep-associated parameters and the following variables: BMI, WC, HC, and WHR. Logistic regression was used to investigate relevant risk factors for moderate-to-severe OSA. The discrimination ability of the fitted logistic models was assessed using the receiver operating characteristic (ROC) curve. The discrimination ability of the model was reported through the area under the estimated ROC curve with 95% confidence intervals (CI). An ROC analysis was also performed to determine the optimal cut-off values for the identifying moderate-to-severe OSA. Considering that age, ESS and the WHR may be closely related to OSA, multiple linear regression models were evaluated to analyze the OSA severity. A *p* value < 0.05 was considered statistically significant.

## Results

### Basic characteristics of participants

Among the 244 included participants, 177 were nonobese patients and 67 were obese patients. Among the four groups of patients, the AHI, HI, LSPO_2_, ODI values were significantly different (*p* < 0.05) (Table 1). There was no obvious difference in AI among groups (Table 1), suggesting that various fat accumulations had a very weak effect on apnea events. In addition, the ESS score, as the indicator of daytime sleepiness, did not differ among these four groups (Table 1), and this may imply that daytime sleepiness was not related to obesity or central fat distribution.

**Table 1:** Demographic and polysomnographic profile of subjects

	Obesity BMI ≥ 28 kg/m^2^		Non-obese BMI < 28 kg/m^2^		*p*-value	*F*
	(A) WHR ≥ 0.9	(B) WHR < 0.9	(C) WHR ≥ 0.9	(D) WHR < 0.9		
AHI (h)	38.1 ± 27.4	38.5 ± 18.9	23.4 ± 16.0^***^	21.5 ± 15.2^***#^	< 0.01	11.60
AI (h)	15.4 ± 25.0	15.9 ± 16.2	10.0 ± 13.9	9.8 ± 13.5	0.16	1.77
HI (h)	22.7 ± 18.6	22.7 ± 8.8	13.3 ± 9.1^***^	12.0 ± 9.0^***#^	< 0.01	12.07
LSPO2 (%)	76.2 ± 11.4	81.3 ± 5.6	83.3 ± 9.3^***^	85.1 ± 7.5^***^	< 0.01	12.35
ODI (h)	39.7 ± 29.1	32.0 ± 17.9	17.3 ± 15.0^***^	15.6 ± 15.8^***^	< 0.01	21.40
AI/HI	2.1 ± 5.4	0.87 ± 1.30	2.37 ± 9.64	3.03 ± 0.01	0.87	0.24
ESS	6.3 ± 5.3	5.9 ± 4.4	7.2 ± 5.1	7.1 ± 4.3	0.64	12.93
Height (cm)	175.4 ± 11.9	176.3 ± 9.2	171.3 ± 6.2	172.4 ± 6.6	0.02	3.45
Weight (kg)	133.5 ± 30.0	106.2 ± 32.7	67.1 ± 7.1	70.8 ± 10.6	< 0.01	188.17
WC (cm)	129.0 ± 18.0	104.8 ± 14.6	90.7 ± 5.1	85.6 ± 7.7	< 0.01	212.06
HC (cm)	127.7 ± 21.3	121.3 ± 19.9	95.8 ± 5.5	99.1 ± 6.6	< 0.01	91.89
WHR	1.04 ± 0.30	0.87 ± 0.04	0.95 ± 0.04	0.86 ± 0.03	< 0.01	19.01
Age (y)	30.24 ± 0.99	39.75 ± 3.61	50.49 ± 1.69	43.41 ± 1.24	< 0.0001	28.76

*** indicated *p* < 0.001 versus group A; ^#^ indicated *p* < 0.05 versus group B

### Correlation analysis between the severity of OSA and WHR

**Fig. 1:**
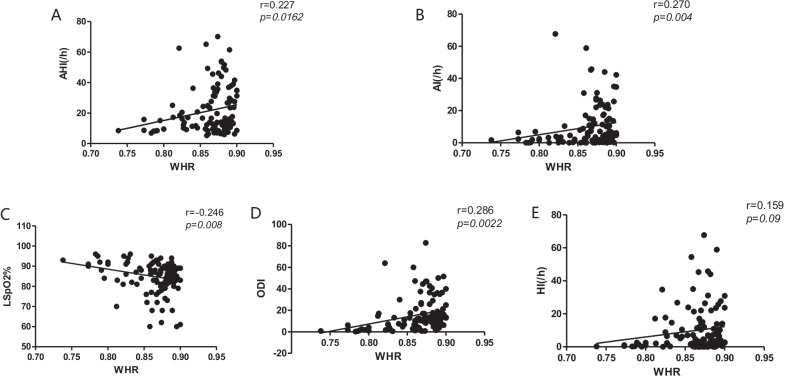
Correlation analysis of WHR with indicators of severity of OSA (including panel **A**: AHI, panel **B**: AI, panel **C**: LSpO_2_, panel **D**: ODI, and panel **E**: HI) in Group D (WHR < 0.9 and BMI < 28 kg/m^2^). AHI: Apnea–hypopnea index; AI: Apnea index; HI: Hypopnea index; LSpO_2_: Lowest oxygen pulse saturation; ODI: The oxygen desaturation index

The OSA severity was evaluated via respiratory events and oxygen desaturation. According to the BMI and WHR, the participants were divided into 4 groups as mentioned before. In Groups A, B and C, no correlations were found between the WHR and sleep severity variables, such as AI, HI, AHI or ODI (Additional file 2: Table S1). A negative correlation between the WHR and LSpO_2_ was observed in Group A but not in Group B and Group C (Additional file 2: Table S1). In Group D, the correlation analysis between WHR and sleep severity variables were shown in Fig. 1. The WHR was positively correlated with AHI (r = 0.227, *p* = 0.0162), AI (r = 0.270, *p* = 0.004) and ODI (r = 0.286, *p* = 0.0022) (Fig. 1 A, B, D). In addition, the WHR was negatively correlated with LpSO_2_ (r =  − 0.246, *p* = 0.008) in Group D (Fig. 1C). There was a marginal correlation between the WHR and HI (r = 0.159, *p* = 0.09) (Fig. 1E). The WHR yielded a moderate correlation with four OSA severity markers (AHI, AI, LSpO_2_ and ODI) but a weak correlation with HI (Fig. 1A–E).

### WHR as an independent risk factor for OSA severity and screening marker for moderate-to-severe OSA in nonobese OSA patients

**Fig. 2:**
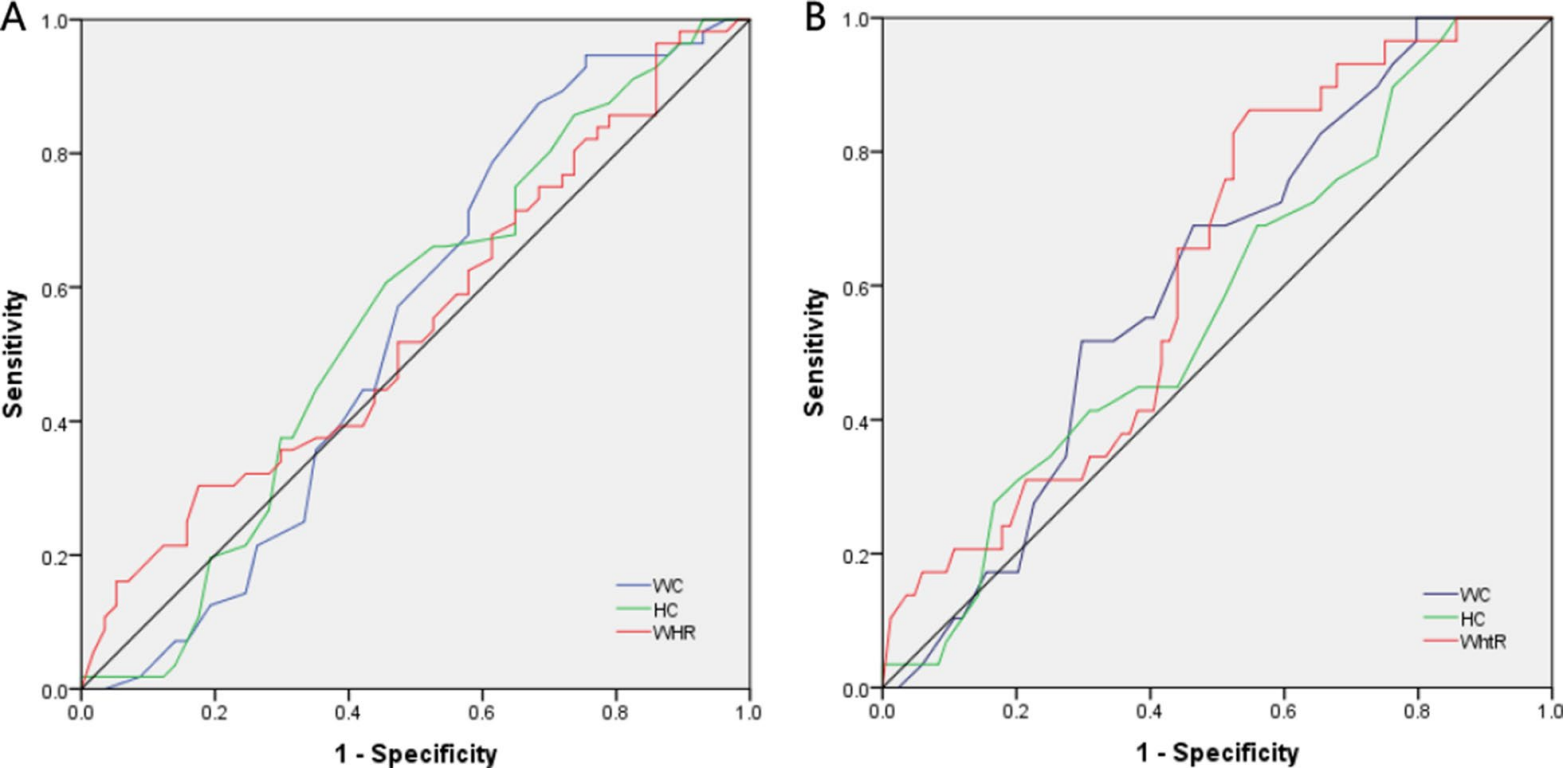
The area under the curve (AUC) of WC, HC, WHR for moderate OSA (**A**) and severe OSA (**B**). See Table 3 for explanations

Multivariate logistic regression modelling was conducted for the 4 groups defined by BMI/WHR status. In each group, weight, BMI, WC, HC, and WHR were tested as predictors of the occurrence of moderate/severe OSA. In Group D, WC, HC, and WHR were found to be independent statistically significant risk factors for moderate OSA, and marginally significant risk factors for severe OSA (*p* < 0.05) (Table 2). Moreover, the odds ratios (OR) of having moderate/severe OSA were higher for the WHR than for the weight, BMI, WC and HC variables (Table 2). In contrast, among the Group A, B and C, the occurrence of moderate or severe OSA was not significantly associated with weight, BMI, WC, HC, or WHR (Table 2). Table 3 showed the area under the curve (AUC) derived using ROC curves for these different parameters to predict moderate or severe OSA. The WHR, in comparison to WC and HC, yielded the highest risk estimates for severe OSA. The AUC for the WHR in identifying severe OSA in Group D was significantly greater than those of WC and HC (Fig. 2). The WHR cut-off point of 0.873, when used to predict severe OSA in Group D, yielded a sensitivity of 65% and specificity of 56% (*p* < 0.05). The WC, weight, HC and WHR had collinearity relationships; therefore, with AHI and ODI set as individual dependent parameters, age, ESS and WHR were subjected to linear regression analyses (Additional file 3: Table S2). The WHR was an independent risk factor for ODI in all subjects and for AHI and ODI in nonobese patients.

**Table 2:** Multivariate logistic regression analysis of the occurrence of moderate/severe OSA

	Obesity BMI ≥ 28 kg/m^2^						Non-obese BMI < 28 kg/m^2^					
	(A) WHR ≥ 0.9			(B) WHR < 0.9			(C) WHR ≥ 0.9			(D) WHR < 0.9		
Parameter	OR value	95% CI	*p*-value	OR value	95% CI	*p*-value	OR value	95% CI	*p*-value	OR value	95% CI	*p*-value
Moderate OSA (AHI ≥ 15 (h))												
Weight (kg)	1.00	0.97–1.03	0.89	1.12	0.58–2.16	0.73	0.99	0.88–1.11	0.80	1.05	0.97–1.15	0.24
BMI (kg/m^2^)	1.12	0.89–1.41	0.34	26,980.9	0–5.896E+28	0.72	1.13	0.66–1.94	0.65	1.10	0.82–1.48	0.53
WC (cm)	0.74	0.19–2.88	0.66	117,756.6	0–1.266E+109	0.92	0.52	0.08–3.38	0.49	0.09	0.01–0.74	0.03^*^
HC (cm)	1.40	0.37–5.36	0.62	0	0–1.551E+86	0.92	1.88	0.31–11.55	0.49	7.55	1.20–47.46	0.03^*^
WHR	4.11E+23	0.00–9.397E+95	0.52	0	0-	0.92	6.629E+24	0.00–1.814E+102	0.53	7.760E+100	2.423E+11–2.486E+190	0.03^*^
Severe OSA (AHI ≥ 30 (h))												
Weight (kg)	1.01	0.98–1.05	0.41	1.09	0.85–1.40	0.52	0.91	0.80–1.03	0.14	1.00	0.91–1.10	0.93
BMI (kg/m^2^)	0.98	0.83–1.16	0.83	0.93	0.29–2.93	0.90	2.12	1.13–4.00	0.02^*^	1.20	0.85–1.70	0.30
WC (cm)	1.22	0.70–2.13	0.49	0.04	0–18,776,422.43	0.75	0.33	0.04–2.52	0.29	0.02	0.00–1.47	0.07
HC (cm)	0.85	0.49–1.47	0.56	14.23	0–433,511,216.0	0.76	3.05	0.42–22.00	0.27	38.73	0.68–2223.264	0.08
WHR	0.00	0–2.642E+26	0.75	2.169E+140	0-	0.79	1.277E + 48	0.000–1.496E+133	0.27	1.120E+191	0.00-	0.07

^*^Indicated *p* < 0.05

**Table 3:** The multivariate logistic regression models for the associations of anthropometric measurements and moderate/severe OSA in group D

	AHI ≥ 15 (h)			AHI ≥ 30 (h)		
	AUC	95% CI	p-value	AUC	95%	p-value
WC (cm)	0.54	0.44–0.65	0.43	0.61	0.50–0.72	0.08
HC (cm)	0.55	0.45–0.66	0.34	0.57	0.46–0.69	0.26
WHR	0.55	0.44–0.65	0.39	0.63	0.52–0.74	0.04*

^*^Indicated *p* < 0.05

## Discussion

It is well known that obesity is a risk factor for moderate-to-severe OSA, but it is often overlooked that nonobese males may have moderate-to-severe OSA. The current study first grouped the recruited patients according to a combination of their BMI and WHR to investigate the relationship between the WHR and the occurrence and severity of OSA. This is the first study to provide evidence that the WHR is positively correlated with OSA severity in nonobese patients. Moreover, a similar correlation was not observed in obesity and/or visceral adipose accumulation (WHR ≥ 0.9) patients. In line with the correlation analysis, WHR was an independent risk factor for the presence of moderate-to-severe OSA only in nonobese patients.

A number of clinical and animal studies have demonstrated a close relationship between OSA and obesity. The BMI was devised in the nineteenth century by Quetelet [16], and is the most widely used technique to diagnose obesity in individual subjects. Many studies reported that BMI was associated with OSA severity [3, 17–19]. Recently, BMI has been more widely regarded as a sole indicator of general adipose tissue. In comparison to subcutaneous obesity, visceral obesity tends to be associated with OSA-associated insulin resistance, dyslipidemia, and glucose intolerance. Increased deposition of fat in the visceral compartment may occur as an unfavorable outcome consequent to saturation of subcutaneous adipose tissue to store fat. Therefore, some surrogates of anthropometric measures for different fat distributions, including the neck circumference (NC), WC, HC, and WHR, have been increasingly studied. In a multiple logistic regression model, BMI (obese vs. nonobese) was not associated with OSA, but a high WHR (at cut-off points of 1 for men and 0.85 for women) was associated with an odds ratio of 2.6 (1.2–5.8) [20]. Another study reported that the WHR was the most reliable correlate of OSA in both sexes. NC was solely an independent risk factor for male OSA patients but not for female OSA patients [21]. Therefore, practical considerations appeared to favor the use of WHR as an alternative to BMI.

According to the WHO Expert Consultation on WC and WHR, the performance of measures such as WC and WHR, used in conjunction with BMI, might contribute to the development of composite indices for use with individuals and the community [22]. To better investigate the effect of fat mass and fat distribution on the presence and severity of OSA, our patients were grouped based on different BMI and WHR degrees. These four groups were individually representative of patients with different obesity statuses: patients with central obesity, patients with noncentral obesity, nonobese patients with central fat and nonobese patients. The current results suggested that the WHR is a better predictor for moderate-to-severe OSA in nonobese patients and is correlated with the severity of OSA. In accordance with previous research, the WHR showed the strongest correlation with AHI in BMI < 25 kg/m^2^ in comparison with BMI, NC, WC and waist-height ratio [23]. Obesity, an established major risk factor for OSA, is less common among Asians, and the reported values of body mass indices (BMIs) of Asians with OSA are lower than those of their Caucasian counterparts [24]. Asian Indians have a high risk of developing OSA with small increments in their upper adiposity, despite having a normal BMI [25]. Our results suggest that in nonobese patients, centrally located rather than peripherally located fat that contributes to the pathogenesis and severity of OSA and is thus especially essential when evaluating OSA risks in nonobese Asian populations.

Accumulating evidence suggests a vicious cycle when obesity and OSA meet. Several mechanisms may account for the increased risks of OSA with obesity. Unexpectedly, our results did not support any significant relationship between the WHR and OSA severity in obese patients, suggesting that there are other mechanisms worth exploring in addition to fat accumulation. In men, for any given BMI quartile, the mean forced expiratory volume in the first second (FEV1) and forced vital capacity (FVC) decreased with increasing WHR. Moreover, the lowest lung function values were observed among those in the top WHR quartile in nonobese patients [26]. Notably, the total lung volume was an important factor affecting the collapse of the upper airway. Decreases in lung volumes likely crippled caudal traction on the upper airway, facilitating its collapse [27]. The above studies further supported our results. The WHR not only affects fat accumulation but also affects the collapsibility of the airway through alternation of the total lung volume, in turn promoting OSA. In our study, although both Groups C and D included the nonobese population, only the comparatively smaller WHR values (Group D) were related to the severity of OSA. This may have been because in the nonobese population, the relationship between WHR and OSA is limited to the lower WHR values; once it exceeds the threshold, the WHRs may plateau with the OSA severity.

Although WHR was originally regarded as an indicator for abdominal obesity, its significance in predicting cardiovascular disease and other chronic complications is receiving increasing attention. An increasing WHR has been found to be associated with an increasing risk of myocardial infarction [28] and heart failure [29] after adjusting for BMI and other risk factors among those regarded as being very-below-normal weight or of normal weight. Men with the highest-quartile of WHR had multivariate-adjusted hazard ratios of 1.81 for total stroke and 2.26 for ischemic stroke compared with men with the lowest quartile of WHR [30]. A decrease in WHR of more than 5% significantly reduced the risk of chronic kidney disease development in nonalcoholic fatty liver disease (NALFD) patients, even in those who were nonobese. Thus, the serial monitoring of the WHR may be prioritized in the management of NAFLD [31]. In addition, the WHR independently predicted mortality and the first cardiovascular event in peritoneal dialysis patients with BMI less than 28 kg/m^2^ after adjustment for known ischemic heart disease [32]. These data provided sound evidence for the strong relationship between WHR and moderate-to-severe OSA risk in nonobese patients.

There are several strengths and limitations in our study. The advantage of this study is that we combined WHR measurements and BMI to identify different OSA phenotypes. Significantly, WHR measurements can be conducted quickly, reliably, non-invasively, and inexpensively, making them the valuable measurements for clinical practice in nonobese patients. Our study, however, has several limitations. First, this is an observational study based on only one center; we analyzed only men, and obesity patterns involve sex-specific characteristic. Second, the WHR consistency requires further assurance, as it was measured by different medical staff members, even though they received standardized training. Third, the normal WHR cut-off value differs with regard to ethnic populations, occupations, education levels, etc. [33]. To date, there are a lack of large-scale epidemiological studies on normal WHR values in the Chinese population. Additionally, we did not have details on the above variables in the whole sample. Fourth, since data on comorbidities and NC are not collected, the significance of exploring anthropometric measures is not sufficiently comprehensive in nonobese people.

## Conclusions

In conclusion, the present study demonstrates that the WHR is associated with the severity of OSA in a nonobese male population. Moreover, the WHR is a screening marker for the presence of moderate-to-severe OSA and an independent risk factor for OSA severity in nonobese male OSA patients. Thus, physicians should measure WHR to help determine the risk of OSA in nonobese male patients. The correlations between the WHR and the risks of cardiovascular and metabolic disorders in nonobese OSA patients need more exploration.

## Supplementary Information

**Additional file 1:** Enrollment flowchart for the study.

**Additional file 2: Table S1.** Correlation analysis of WHR and OSA severity in group A, B and C.

**Additional file 3: Table S2.** Coefficients of multiple linear regression analysis on AHI and ODI.

## Data Availability

The datasets used in the current research are available from the corresponding author upon reasonable request.

## Abbreviations

OSA: Obstructive sleep apnea
BMI: Body mass index
AHI: Apnea–hypopnea index
PSG: Polysomnography
WHR: Waist-hip ratio
PET-CT: Positron emission tomography-computed tomography
ABPM: Ambulatory blood pressure monitoring
TST: Total sleep time
HST: Home sleep test
ESS: Epworth Sleepiness Scale
WC: Waist circumference
HC: Hip circumference
AI: Apnea index
HI: Hypopnea index
LSpO_2_: Lowest oxygen pulse saturation
ODI: Oxygen desaturation index
SD: Standard deviation
ROC: Receiver operating characteristic
CI: Confidence intervals
AUC: Area under the curve
NC: Neck circumference
BMIs: Body mass indices
FEV1: Forced expiratory volume in the first second
FVC: Forced vital capacity
NAFLD: Nonalcoholic fatty liver disease
OR: Odds ratios

## References

[1] Guilleminault C, Quo SD (2001). Sleep-disordered breathing: a view at the beginning of the new Millennium. Dent Clin North Am.

[2] Young T, Palta M, Dempsey J, Skatrud J, Weber S, Badr S (1993). The occurrence of sleep-disordered breathing among middle-aged adults. N Engl J Med.

[3] Ip MS, Lam B, Lauder IJ, Tsang KW, Chung KF, Mok YW, Lam WK (2001). A community study of sleep-disordered breathing in middle-aged Chinese men in Hong Kong. Chest.

[4] Redolfi S, Arnulf I, Pottier M, Lajou J, Koskas I, Bradley TD, Similowski T (2011). Attenuation of obstructive sleep apnea by compression stockings in subjects with venous insufficiency. Am J Respir Crit Care Med.

[5] Hanly PJ, Pierratos A (2001). Improvement of sleep apnea in patients with chronic renal failure who undergo nocturnal hemodialysis. N Engl J Med.

[6] Lowe AA, Santamaria JD, Fleetham JA, Price C (1986). Facial morphology and obstructive sleep apnea. Am J Orthod Dentofacial Orthop.

[7] Seto BH, Gotsopoulos H, Sims MR, Cistulli PA (2001). Maxillary morphology in obstructive sleep apnoea syndrome. Eur J Orthod.

[8] Canapari CA, Hoppin AG, Kinane TB, Thomas BJ, Torriani M, Katz ES (2011). Relationship between sleep apnea, fat distribution, and insulin resistance in obese children. J Clin Sleep Med.

[9] Glicksman A, Hadjiyannakis S, Barrowman N, Walker S, Hoey L, Katz SL (2017). Body fat distribution ratios and obstructive sleep Apnea severity in youth with obesity. J Clin Sleep Med.

[10] Tchernof A, Despres JP (2013). Pathophysiology of human visceral obesity: an update. Physiol Rev.

[11] McQuaid SE, Humphreys SM, Hodson L, Fielding BA, Karpe F, Frayn KN (2010). Femoral adipose tissue may accumulate the fat that has been recycled as VLDL and nonesterified fatty acids. Diabetes.

[12] Lam JC, Ip MS (2009). Obstructive sleep apnea and the metabolic syndrome. Expert Rev Respir Med.

[13] Seidell JC (2010). Waist circumference and waist/hip ratio in relation to all-cause mortality, cancer and sleep apnea. Eur J Clin Nutr.

[14] Cairns A, Wickwire E, Schaefer E, Nyanjom D (2014). A pilot validation study for the NOX T3(TM) portable monitor for the detection of OSA. Sleep Breath.

[15] Berry RB, Budhiraja R, Gottlieb DJ, Gozal D, Iber C, Kapur VK, Marcus CL, Mehra R, Parthasarathy S, Quan SF (2012). Rules for scoring respiratory events in sleep: update of the 2007 AASM manual for the scoring of sleep and associated events: deliberations of the sleep Apnea definitions task force of the American Academy of sleep medicine. J Clin Sleep Med.

[16] Eknoyan G (2008). Adolphe Quetelet (1796–1874)–the average man and indices of obesity. Nephrol Dial Transpl.

[17] Ip MS, Lam B, Tang LC, Lauder IJ, Ip TY, Lam WK (2004). A community study of sleep-disordered breathing in middle-aged Chinese women in Hong Kong: prevalence and gender differences. Chest.

[18] Kim J, In K, Kim J, You S, Kang K, Shim J, Lee S, Lee J, Lee S, Park C (2004). Prevalence of sleep-disordered breathing in middle-aged Korean men and women. Am J Respir Crit Care Med.

[19] Suwanprathes P, Won C, Komoltri C, Nana A, Kotchabhakdi N, Guilleminault C (2010). Epidemiology of sleep-related complaints associated with sleep-disordered breathing in Bangkok, Thailand. Sleep Med.

[20] Martinez-Rivera C, Abad J, Fiz JA, Rios J, Morera J (2008). Usefulness of truncal obesity indices as predictive factors for obstructive sleep apnea syndrome. Obesity (Silver Spring).

[21] Lim YH, Choi J, Kim KR, Shin J, Hwang KG, Ryu S, Cho SH (2014). Sex-specific characteristics of anthropometry in patients with obstructive sleep apnea: neck circumference and waist-hip ratio. Ann Otol Rhinol Laryngol.

[22] Nishida C, Ko GT, Kumanyika S (2010). Body fat distribution and noncommunicable diseases in populations: overview of the 2008 WHO expert consultation on waist circumference and waist-hip ratio. Eur J Clin Nutr.

[23] Kim JH, Koo YC, Cho HJ, Kang JW (2018). Relationship between various anthropometric measures and apnea-hypopnea index in Korean men. Auris Nasus Larynx.

[24] Lam B, Lam DC, Ip MS (2007). Obstructive sleep apnoea in Asia. Int J Tuberc Lung Dis.

[25] Snehalatha C, Viswanathan V, Ramachandran A (2003). Cutoff values for normal anthropometric variables in asian Indian adults. Diabetes Care.

[26] Canoy D, Luben R, Welch A, Bingham S, Wareham N, Day N, Khaw KT (2004). Abdominal obesity and respiratory function in men and women in the EPIC-Norfolk Study, United Kingdom. Am J Epidemiol.

[27] Van de Graaff WB (1988). Thoracic influence on upper airway patency. J Appl Physiol.

[28] Murray S (2006). Is waist-to-hip ratio a better marker of cardiovascular risk than body mass index?. CMAJ.

[29] Gao F, Wan J, Xu B, Wang X, Lin X, Wang P (2020). Trajectories of waist-to-hip ratio and adverse outcomes in heart failure with mid-range ejection fraction. Obes Facts.

[30] Hu G, Tuomilehto J, Silventoinen K, Sarti C, Mannisto S, Jousilahti P (2007). Body mass index, waist circumference, and waist-hip ratio on the risk of total and type-specific stroke. Arch Intern Med.

[31] Chon YE, Kim HJ, Choi YB, Hwang SG, Rim KS, Kim MN, Lee JH, Ha Y, Lee MJ (2020). Decrease in waist-to-hip ratio reduced the development of chronic kidney disease in non-obese non-alcoholic fatty liver disease. Sci Rep.

[32] Su WS, Clase CM, Brimble KS, Margetts PJ, Wilkieson TJ, Gangji AS (2010). Waist-to-hip ratio, cardiovascular outcomes, and death in peritoneal dialysis patients. Int J Nephrol.

[33] Fauziana R, Jeyagurunathan A, Abdin E, Vaingankar J, Sagayadevan V, Shafie S, Sambasivam R, Chong SA, Subramaniam M (2016). Body mass index, waist-hip ratio and risk of chronic medical condition in the elderly population: results from the Well-being of the Singapore Elderly (WiSE) Study. BMC Geriatr.

