# Supplementary material for: Waist-hip ratio is an independent predictor of moderate-to-severe OSA in nonobese males: a cross-sectional study
Source: BMC Pulm Med. 2022 Apr 22;22:151. doi: 10.1186/s12890-022-01886-3 (PMC9034636; doi:10.1186/s12890-022-01886-3)
Supplement: Supplementary file 1 — Additional file 1: Enrollment flowchart for the study. [file 12890_2022_1886_MOESM1_ESM.pdf]

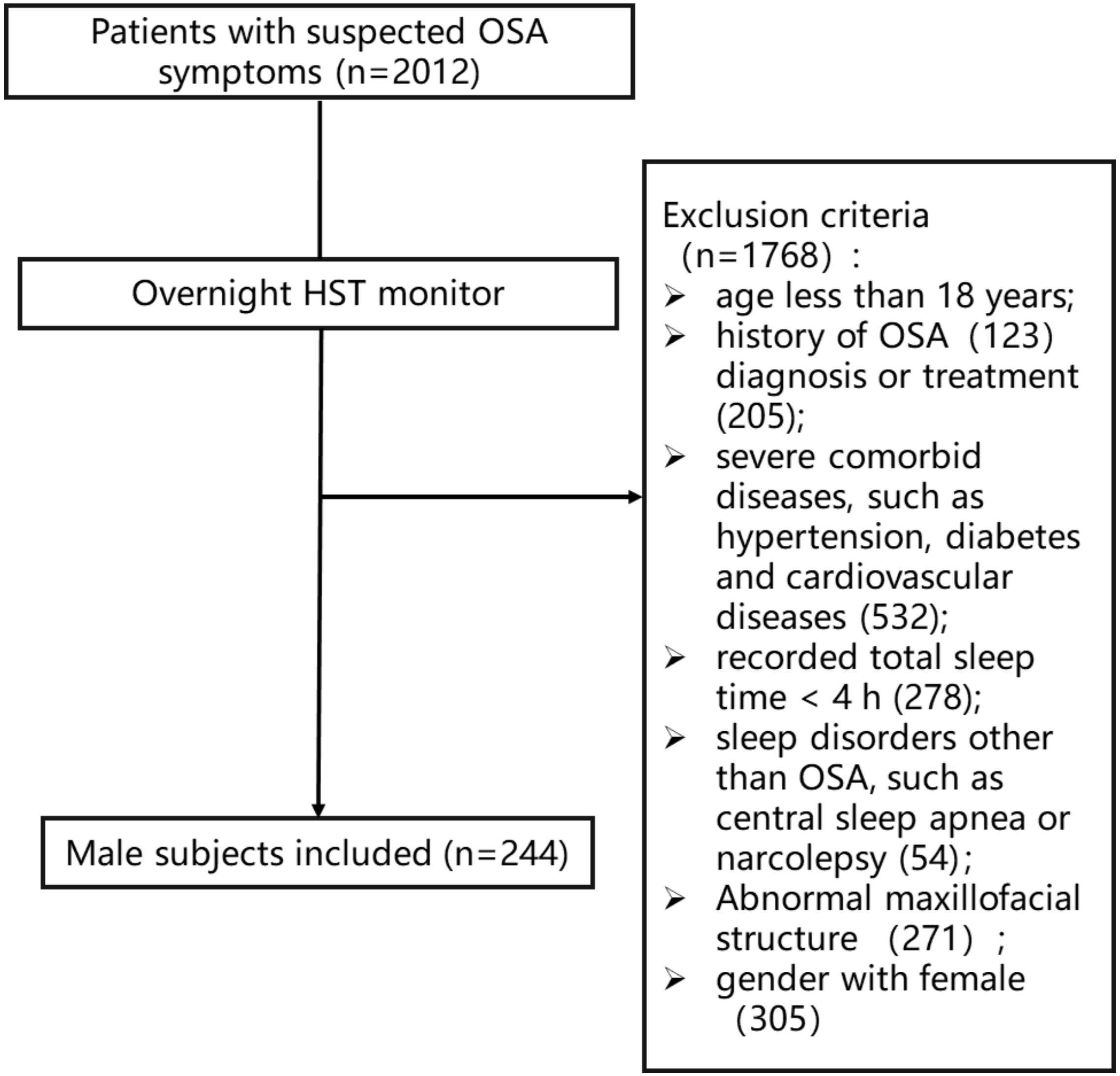

Supplementary Figure: Enrollment flowchart for the study. A total of 2012 suspicious patients with OSA were consecutively recruited from January 2018 to December 2019; 244 patients who met the inclusion criteria were enrolled in the study.
