# Supplementary material for: Waist-hip ratio is an independent predictor of moderate-to-severe OSA in nonobese males: a cross-sectional study
Source: BMC Pulm Med. 2022 Apr 22;22:151. doi: 10.1186/s12890-022-01886-3 (PMC9034636; doi:10.1186/s12890-022-01886-3)
Supplement: Supplementary file 2 — Additional file 2: Table S1. Correlation analysis of WHR and OSA severity in group A, B and C. [file 12890_2022_1886_MOESM2_ESM.docx]

Supplementary table 1. Correlation analysis of WHR and OSA severity in group A, B and C

| Sleep Parameters | Group A | | Group B | | Group C | |
| --- | --- | --- | --- | --- | --- | --- |
|  | WHR | | WHR | | WHR | |
|  | Correlation  coeﬃcient | p-value | Correlation  coeﬃcient | p-value | Correlation  coeﬃcient | p-value |
| AHI (/h) | 0.14 | 0.30 | -0.001 | 0.99 | -0.004 | 0.97 |
| AI (/h) | 0.13 | 0.36 | 0.29 | 0.39 | -0.71 | 0.55 |
| HI (/h) | 0.04 | 0.77 | -0.50 | 0.12 | 0.10 | 0.41 |
| AI/HI | 0.24 | 0.08 | 0.11 | 0.74 | -0.009 | 0.94 |
| LSPO_2_(%) | -0.27^*^ | 0.04 | 0.12 | 0.72 | 0.05 | 0.65 |
| ODI (/h) | 0.12 | 0.39 | -0.21 | 0.55 | -0.38 | 0.75 |

* indicated p<0.05.
