# Supplementary material for: Waist-hip ratio is an independent predictor of moderate-to-severe OSA in nonobese males: a cross-sectional study
Source: BMC Pulm Med. 2022 Apr 22;22:151. doi: 10.1186/s12890-022-01886-3 (PMC9034636; doi:10.1186/s12890-022-01886-3)
Supplement: Supplementary file 3 — Additional file 3: Table S2. Coefficients of multiple linear regression analysis on AHI and ODI. [file 12890_2022_1886_MOESM3_ESM.docx]

Supplementary table 2: Coefficients of multiple linear regression analysis on AHI and ODI

|  | Independent variable | Standardized  coefficient | t | p-value | Standardized  coefficient | t | p-value |
| --- | --- | --- | --- | --- | --- | --- | --- |
|  |  | Dependent variable AHI (/h) | | | Dependent variable ODI (/h) | | |
| All subjects (A-D) | Age (y) | -0.06 | -0.98 | 0.33 | -0.12 | -1.92 | 0.06 |
|  | ESS | 0.07 | 1.15 | 0.25 | 0.09 | 1.37 | 0.17 |
|  | WHR | 0.09 | 1.46 | 0.15 | 0.13 | 2.05 | *0.04 |
| Group D ( WHR < 0.9 and BMI < 28kg/m²) | Age (y) | 0.005 | 0.05 | 0.96 | 0.03 | 0.35 | 0.73 |
|  | ESS | 0.02 | 0.18 | 0.86 | 0.07 | 0.80 | 0.425 |
|  | WHR | 0.24 | 2.44 | *0.02 | 0.29 | 3.08 | *0.03 |

* indicated p<0.05.
